# A Critical Review of the Impact of Sarcoma on Psychosocial Wellbeing

**DOI:** 10.1155/2019/9730867

**Published:** 2019-02-17

**Authors:** Lesley Storey, Lorna A. Fern, Ana Martins, Mary Wells, Lindsey Bennister, Craig Gerrand, Maria Onasanya, Jeremy S. Whelan, Rachael Windsor, Julie Woodford, Rachel M. Taylor

**Affiliations:** ^1^School of Psychology, Queens University Belfast, Belfast BT7 1NN, UK; ^2^Cancer Division, University College London Hospitals NHS Foundation Trust, London NW1 2PG, UK; ^3^Nursing Directorate, Imperial College Healthcare NHS Trust, Charing Cross Hospital, London W6 8RF, UK; ^4^Sarcoma Unit, The Royal National Orthopaedic Hospital NHS Trust, Stanmore, Middlesex HA7 4LP, UK

## Abstract

**Background:**

Previous reviews of outcomes in specific sarcoma populations suggest patients have poor quality of life. In most of these reviews, there is a predominant focus on physical function rather than psychosocial outcome. The aim of this review was to describe the psychosocial impact of diagnosis and treatment on patients with all types of sarcoma.

**Methods:**

Searches were conducted through six electronic databases for publications of any study design using a validated patient-reported outcome measure reporting the psychosocial impact in this population.

**Results:**

Eighty-two studies fulfilled the inclusion criteria. Most (65%) were assessed of being of reasonable quality. The most common aspect of psychosocial wellbeing measured was quality of life (80%). Due to the heterogeneity of methods, outcomes, and populations, it was not possible to make definitive conclusions. It seems there is an improvement in the physical aspects of quality of life over time but not in psychosocial function or mental health. There was no change in mental health scores, but patients reported an improvement in adjusting to normal life. There are no differences according to the type of surgery patients receive, and psychosocial outcomes tend to be poorer than the general population. There is no consistency in identifying the factors that predict/influence psychosocial wellbeing.

**Conclusion:**

The published literature does not provide a clear understanding of the impact of sarcoma diagnosis and treatment on psychosocial wellbeing. Instead, the review demonstrates a need for well-designed studies in this area and a more consistent approach to the measurement of patient-reported outcomes, which include psychosocial domains. Recommendations for future research have been proposed.

## 1. Introduction

Measurement of patient-reported outcome (PRO) and experience has become commonplace in healthcare to measure the quality and impact of healthcare interventions. The phrase “patient-reported outcome” is loosely defined as the report of a health outcome made directly by the patient (rather than an assessment by the healthcare team) [1]. PROs include measures of quality of life (QOL), aspects of mental health, or assessment of physical function and symptoms, such as pain.

The value of measuring and reporting PROs and experience can be seen through nationally collected metrics in several countries [2]. In England, PROs for patients undergoing five surgical procedures have been collected since 2010 [3] with the aim on informing changes to the delivery of care to improve outcome, although there is some debate on how well this has been achieved [4, 5]. Similar benefits have been shown by measuring experience through the National Cancer Patient Experience Survey (NCPES). This has been conducted annually since 2010 and has been invaluable for informing changes to improve care [6, 7]. The survey includes patients with all cancer types but consistently over the last 7 years, patients with sarcoma have generally reported poorer care experience than those with other diagnoses. To further understand the reason for this, a sarcoma-specific experience survey was administered to participants of the 2014/15 NCPES [8]. Patients with sarcoma had a prolonged period to diagnosis, most were treated in multiple hospitals, and many reported experiencing side-effects of treatment, predominantly fatigue [8]. Furthermore, the results indicated that having a written treatment plan was more significant to a better experience than having a clinical nurse specialist (shown to be the most important factor for a good experience of patients with other cancers in the main NCPES).

While this survey elicited greater understanding of the experience of processes of care, it did not tell us about the outcomes. There have been a number of studies exploring QOL after a diagnosis of sarcoma, and the results of many of which have been presented in previous reviews [9–15]. These focus on specific populations, such as bone/extremity sarcoma to compare different surgical techniques [9–11, 13, 14], soft tissue sarcoma [15], and gastrointestinal stromal tumours (GIST) [12]. In most of these reviews, there is a predominant focus on physical function and its objective measurement rather than QOL or psychosocial outcome. Furthermore, despite reviews having similar target populations [10, 14] or inclusion criteria [9, 11, 13], there is a disparity in the studies included in these reviews, with none seeming to include all potentially relevant studies. These reviews consistently indicate that patients with sarcoma have poorer physical function than the general population and other types of cancer, probably associated with a high degree of disability. It is interesting to note that, despite poor physical function and disability, these reviews suggest no negative influence on emotional or social function. This is in contrast to what has been reported in the few qualitative studies that provide an in-depth description of the experience of living with a sarcoma diagnosis, that show the impact and challenges that treatment has on body image, self-esteem, mental health [16, 17], ability to work, and participation in social activities [16, 18–20].

To gain a greater understanding of the impact of sarcoma on patients' psychosocial wellbeing, a more detailed review is therefore indicated. The aim of this review was to describe the impact of the diagnosis and treatment of all types of sarcoma on psychosocial wellbeing, in patients of all ages, undergoing all types of treatment. Psychosocial wellbeing was defined broadly as “the way a person thinks and feels about themselves and others, including being able to adapt and deal with daily challenges while leading a fulfilling life (e.g. this included measurements of quality of life, anxiety, coping, social support but excluded clinical/medical outcomes, such as toxicity, and adherence)” [21]. Specific objectives were to

1. Identify published research on patients' psychosocial wellbeing using validated PRO measures
2. Describe psychosocial wellbeing
3. Identify psychosocial interventions that have been developed and evaluated to improve psychosocial wellbeing
4. Determine which factors influence or predict psychosocial wellbeing
5. Make recommendations for future research and clinical practice

## 2. Methods

### 2.1. Data Sources and Search Strategy

The literature review was guided by search terms used previously in reviews of patient-reported and psychosocial outcomes [10, 21, 22]. The search was conducted on the following electronic databases up until December 2017: BNI (British Nursing Index), Medline, PsycINFO, CINAHL (Cumulative Index to Nursing and Allied Health Literature), AMED (Allied and Complementary Medicine), and ASSIA (Applied Social Sciences Index and Abstracts). Selected journals were hand searched to ensure relevant references were not missed in the electronic search.

The search terms included population (sarcoma, bone tumour, and gastrointestinal stromal tumour) and terms reflecting psychosocial outcomes (quality of life, psychological wellbeing, and social function). The search used both text words and Medical Subject Headings (MeSH) terms (Supplementary Materials, Table A1).

### 2.2. Eligibility Criteria and Study Selection

Studies were eligible for inclusion in the review if they

- Reported a primary or secondary PRO related to psychosocial wellbeing, evaluated through reporting results from a validated measure
- Used a quantitative study design
- Published in English, Spanish, or Portuguese in a peer-reviewed publication

Studies were excluded if they

- Did not have a validated patient-reported outcome measure
- Included groups other than sarcoma patients (e.g., partners, parents, friends, healthcare professionals, etc.) unless the results of sarcoma patients were reported independently
- Included patients with a diagnosis other than sarcoma, unless the results for the sarcoma population were reported independently
- Focused solely on Kaposi Sarcoma

An initial screening of the search results based on titles and abstracts was conducted by one reviewer, and a second reviewer independently screened 10%. The full texts of potentially eligible studies were obtained and information from each study was extracted directly by four reviewers (15–30 papers each) into a data extraction file on Microsoft Excel to ensure consistent information was recorded from all studies. Where a study was suspected of not being eligible, the full text was independently reviewed by another team member before exclusion.

### 2.3. Methodological Quality

There is no critical appraisal tool specific for survey studies, only for the use of patient-reported outcome measures in randomised controlled trials [23], so review-specific criteria were established, based on the CONSORT PRO guidance [24] and recommendations for good practice in survey methods [25] (Table 1). The percentage of criteria that were fulfilled was calculated for each study and independently checked by a separate member of the review team. Studies were classified as Q1 (achieved >75% of quality criteria), Q2 (fulfilled 50–74% of quality criteria), or Q3 (<50% of quality criteria achieved) [26, 27].

### 2.4. Method of Synthesis

Two reviewers independently reviewed the results of the included studies. Due to the heterogeneity of participants, measures, and methods, it was not possible to conduct any meta-analysis, so results were summarised descriptively, and where a comparator was used (either reference group, healthy control, or other cancer population), this was tabulated to show whether it was better (+), worse (−), or no different (=). Factors influencing PRO were identified and tabulated according to the frequency with which each factor was reported.

## 3. Results

### 3.1. Objective 1 : Identify Published Research on Patients' Psychosocial Wellbeing Using Validated PRO Measures

**Figure 1:**
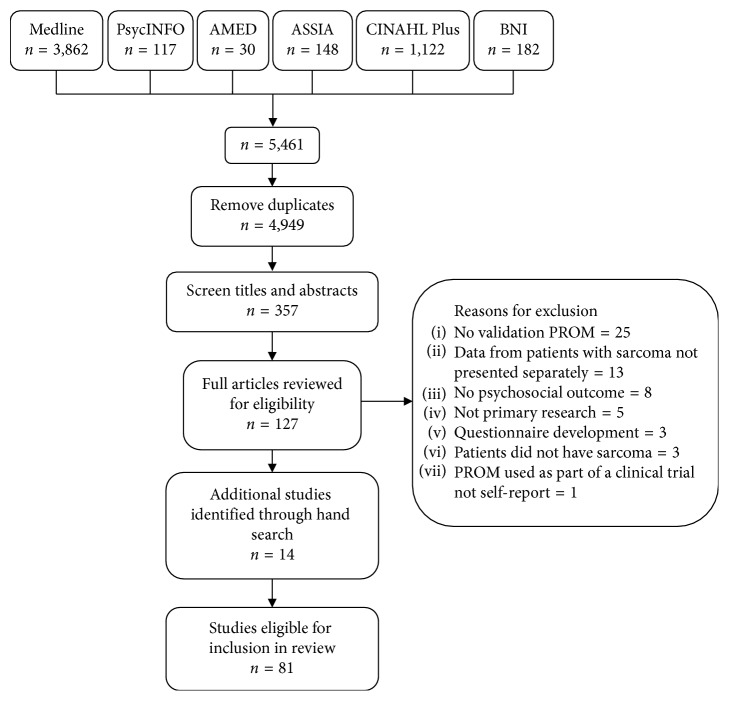
Results of the search strategy. AMED: Allied and Complementary Medicine; ASSIA: Applied Social Sciences Index and Abstracts; BNI: British Nursing Index; CINAHL: Cumulative Index to Nursing and Allied Health Literature; PROM: patient-reported outcome measure.

The search identified 5,461 papers, of which 141 were reviewed in full and 81 were eligible for inclusion [9, 28–107] (Figure 1). Research on psychosocial outcomes had been conducted for over 35 years. Most studies had been conducted in Europe (*n* = 37) or North America (*n* = 35). The majority of studies were single centre (*n* = 52) and focused on investigating outcomes when active treatment had ended (*n* = 59; Table 2). The majority (*n* = 65; 80%) were observational studies although one paper reported QOL as part of a clinical trial of an investigational medicinal product. Data from this paper were included as they were presented as observational [94]. Most studies were good/reasonable quality (rated Q1 and Q2, *n* = 53), but 28 were of poor quality (rated Q2/3 and Q3) (Supplemental file, Table A2). The most common omissions were as follows: not reporting how missing data were handled (*n* = 74); not presenting a comparison of the demographic characteristics of nonparticipants (*n* = 59); not stating how the measure was administered (*n* = 36); and not giving details of how the scores were interpreted (*n* = 28; Table A2).

Studies included between 1 and 6 measures with 34 reporting use of a single measure (Supplemental file, Table A3). The most commonly measured psychosocial outcomes were QOL (*n* = 65) and aspects of mental health (*n* = 28), but other outcomes included self-worth (*n* = 8); social support (*n* = 5); adjustment to normal life (*n* = 4); coping, body image, fatigue, and satisfaction with life (*n* = 3 each); sexual function (*n* = 2); and resilience, fear of recurrence, optimism, social wellbeing, family function, expectations for the future, and benefit finding (*n* = 1 each). While there were 64 different patient-reported outcome measures (with most studies using multiple measures), the most common were SF-36 (*n* = 31) and QLQ-C30 (*n* = 16). Ten studies used a QOL measure that could give a total and/or broad domain summary scores (such as physical or mental component score), but these results were not reported [41, 43, 44, 49, 53, 64, 74, 91, 99, 101].

Participant characteristics are presented in Table 3. In summary, psychosocial outcomes have been measured in 8,823 patients, with a sample size ranging from 10 to 1094 per study (response rate median 76%, range 13–100%). It was not possible to calculate the response rate in 12 studies due to a lack of reported information. The majority of studies included patients with bone tumours (*n* = 51) and lower limb/extremity tumours (*n* = 47). Thirty-five studies included adults only, four focused solely on children (participants aged less than 18 years), and 39 included children and adults. The age of participants was not reported in three studies.

### 3.2. Objective 2: Describe Psychosocial Wellbeing

A summary of all the results is presented in the supplemental file (Table A4). A number of papers reported findings which were unsurprising, including that patients who experienced higher pain also had lower psychological outcomes [104], patients with higher anxiety and depression had greater fear of recurrence [37], those who were distressed had lower QOL and had more shame and stigma than those without distress [97], and those with severe fatigue had lower QOL and self-efficacy compared to those with nonsevere fatigue [79].

Thirteen studies used a longitudinal design to compare between different phases of the cancer timeline (Table 4). Results suggest that there is an improvement in the physical aspects of QOL over time but not psychosocial function or mental health. There was no change in mental health scores [73, 75], but patients reported an improvement in adjusting to normal life [38] (Table 4). Twenty-three studies reported outcomes of a comparison of different types of treatment, e.g., limb salvage surgery versus amputation (Table 5). While there were some reports of amputations being associated with a poor outcome [41], the majority showed no difference. Similarly, there were no differences in the comparison of outcome in patients who had limb-sparing surgery, amputation, and rotationplasty, although there was one report of better role function for patients with rotationplasty compared to those with limb-sparing surgery [56]. Other psychosocial outcomes that were measured mostly showed no difference according to type of surgery, although patients who had amputations were shown to have poorer mental health [30, 36] but better feelings of self-worth [30] (Table 5).

Twenty-six studies compared QOL scores to reference values, either general population data provided with the measure or noncancer control data collected as part of the study (Table 6). Six studies found no differences in QOL [51, 59, 62, 78, 85, 103] and 15 reported that patients with sarcoma had poorer QOL, mostly in the physical domains only [29, 43, 44, 47–49, 61, 63, 64, 74, 81, 99–101], but three studies found patients with sarcoma had better QOL in the psychosocial domains [34, 49, 53]. One study was not able to make any conclusions because it used three measures of QOL, which all gave different results [31] (Table 6). In comparison to patients with other types of cancer, those with sarcoma reported similar levels of fatigue [29] but poorer mental health [71, 72, 78]. Aksnes et al. [29] and Hind et al. [57] reported QOL being poorer in those with sarcoma in contrast to Ostacoli et al. [72] and Podleska et al. [78] who found better QOL (Table 7).

Focusing on the most commonly used measures, results produced by the SF-36 (Table 8) indicated that there was no difference in QOL between amputation, limb-sparing surgery, and rotationplasty [28, 31, 44, 45, 52, 60], and QOL was poorer than reference values [29, 34, 44, 47–49, 63, 64, 81, 99, 101]; patients with sarcoma had poorer physical function in comparison to patients with other cancer types [29] and an improvement in QOL over time [33, 55, 83] (Table 8). QOL measured by the QLQ-C30 (Table 9) indicated no difference between amputation and limb-sparing surgery [31, 107], but patients with rotationplasty had better role function [56]. A greater number of studies showed no difference to reference values [59, 78, 85] and poorer QOL in patients with sarcoma [74, 101] (Table 9). Four studies used both the SF-36 and the QLQ-C30; results were comparable in two [79, 91], whereas Veenstra et al. [101] noted no difference in comparison to the general population with the QLQ-C30 but significant difference in SF-36 scores. Likewise, Barrera et al. [31] found significantly poorer SF-36 Physical Component Scores but similar Mental Component Scores in comparison to the reference value. However, results using the QLQ-C30 indicated patients with sarcoma had significantly better Global Health Status, Role Function, Emotional Function, and Social Function than the reference value and similar physical function.

Mostly there were no differences in other aspects of psychosocial outcome that were measured such as social support, body image, and self-worth. However, patients reported having better expectations for the future and greater satisfaction with leisure compared to the general population.

### 3.3. Objective 3: Identify Psychosocial Interventions That Have Been Developed and Evaluated to Improve Psychosocial Wellbeing

While psychosocial measures were identified as being secondary end points in a number of clinical trials (not included in this review), no psychosocial interventions specific to patients with sarcoma were identified to improve PRO.

### 3.4. Objective 4: Determine Which Factors Influence Psychosocial Wellbeing

Twenty-three studies conducted analysis to identify factors that could predict aspects of psychosocial wellbeing. Factors predicting QOL included disease-related variables, gender, age at the time of diagnosis/study, level of education, employment and marital status, body image, everyday competence, physical function, recurrence of disease, and symptom distress [29, 38, 40, 44, 47, 50, 57, 66, 69, 73, 81, 87, 89, 93, 96]. Severe fatigue was influenced by disease-related variables, optimism, physical function, and psychological distress [79, 90]. General psychosocial outcomes (including mental wellbeing and posttraumatic growth) were associated with age at the time of diagnosis/study, gender, marital status, disease-related variables, time since treatment ended, coping, and social support [36, 67, 73–76, 106]. While these factors were shown to predict PRO in some studies, this was not always the case. For example, age at diagnosis/study, gender, time since treatment, level of education, recurrence, and physical function were also shown *not* to be predictive of outcome [36, 40, 69, 89, 90, 93].

Due to the huge variation in outcomes, measures, population, and methods used, it was not possible to explore in any detail or make conclusion about what might influence or predict psychosocial wellbeing.

Interestingly, while there has been much work comparing between different types of surgery, there has been little exploration of differences according to type of sarcoma. A number of studies included patients with multiple cancer types [35, 41, 47, 50, 54, 67, 73–76, 90, 97, 102, 104], but the only direct comparisons were made by Chan et al. [35] who reported patients with GIST had better QOL and mental health compared to those without GIST, and patients with giant cell tumours had poorer quality of life compared to those with osteosarcoma and chondrosarcoma [91]. Similarly, Marina et al. [67] identified type of diagnosis as being an influencing factor for anxiety, showing patients with Ewing sarcoma had a relative risk of anxiety double that of patients with soft tissue sarcoma. However, other studies showed type of diagnosis was not found to influence psychosocial outcomes [73–76].

## 4. Discussion

This review aimed to collate all studies reporting psychosocial wellbeing using a valid measure, in patients with sarcoma. Overall it seems there is an improvement in the physical aspects of QOL over time but not in psychosocial function or mental health. Psychosocial wellbeing is poorer than the general population, and there is no difference if patients have amputation, limb-sparing surgery, or rotationplasty. However, results are not conclusive and, due to a number of factors, must be viewed with caution. The methodological quality of many studies was poor, especially in the selection and administration of outcome measures; even those rated “high quality” using our prespecified criteria reported some significant limitations. For example, Hinds et al. [58] used the PedsQL, a well-established, validated measure of QOL for children and adults, but in their study of adolescent QOL they noted low internal consistency in the social function domain so were unable to report these results. This also limited their ability to report an aggregate psychosocial domain and overall QOL score.

Incomplete reporting of QOL data was noted in a number of papers where the authors did not present total, summary, and domain scores [40, 44, 64, 99, 101]. While the level of reporting depends on the aims of the study, if the aim, as in the majority of the included studies, was to report QOL, then domain as well as summary/total scores can help to identify which aspects of life are better/worse than the comparator. The lack of detail on how a measure was administered was also a considerable problem. Our minimum criteria of quality was the mode of administration; if we had included a criteria of the precise detail of administration (including who, where, and how), then more studies would have been judged as poor quality. Such information is likely to help the reader to judge the degree of bias and how the administration of questionnaires could have influenced the results [24]. Finally, if the item scores are combined to make an overall aggregated score without appropriate imputation, then the overall score could be erroneously low.

Another problem with assessing PRO in patients with sarcoma is the heterogeneity of the population, both in terms of age, disease type, and anatomic location. Sarcoma affects children, adolescents, and adults, and a number of studies used measures which had not been validated for that age group. This was especially an issue with studies using the SF-36 and QLQ-C30, which are only validated for patients aged 18 onwards but 43% included participants younger than 18 years old. Measures developed for adults may not be specific enough to detect QOL differences in children and adolescents. The lack of measures that can span the full age range of a sarcoma population is a well-recognised limitation of PRO research in adolescents and young adults with cancer, especially with the content of current generic measures not reflecting issues important to young people [1, 109–111].

A further factor impacting the results in the current review has been the use of generic population or generic cancer measures of QOL. The need for disease-specific measures is well recognised as having the sensitivity to detect changes related to a particular condition [112]. Quality of life measures for various cancer types have been developed (for example, see http://qol.eortc.org/questionnaires/). The lack of difference between a sarcoma population and general population may not be detected because the content of the measure may not reflect the specific challenges related to having a sarcoma diagnosis. The fact that there are questionnaires specific for other cancer types supports the need for content reflecting tumour-specific experience. This was highlighted in a study by Skalicky et al. [113] who showed the uniqueness of sarcoma in the development of the Soft Tissue Sarcoma Symptom Inventory; clinicians and patients identified eight important symptoms not reflected in existing measures (including the SF-36 and QLQ-C30). If a measure does not reflect the experience of the population, then it is unlikely that it will detect important differences.

The size of the studies in this review also compromised our ability to conduct any statistical analysis of the results. Most of the identified studies had small samples, with less than a quarter including more than a hundred patients, and half including less than fifty patients. Sample size was also a particular issue for studies aiming to identify influencing or predictive factors that included large numbers of variables; these were potentially underpowered to be able to identify anything of significance.

## 5. Conclusion

Unfortunately, the results of the studies included in this review do not provide us with a clear understanding of the impact of sarcoma on psychosocial outcomes. Instead, the review demonstrates that there is a need for well-designed studies in this area and a more consistent approach to the measurement of patient-reported outcomes. It is clear that sarcoma has an impact on psychosocial wellbeing, but we do not know enough about what aspects are impacted, and at what point in the patients diagnostic trajectory.

We make a number of recommendations based on this review: first, more detailed understanding of patients' experience of being diagnosed and living with sarcoma is needed, so similarities and differences between sarcoma-related variables (at a minimum, type of sarcoma) can be identified. Second, outcome measures which reflect the particular physical and psychosocial concerns and experiences of patients with sarcoma need to be developed. Third, in order to achieve the second recommendation, a large qualitative study is required including patients across ages, types and sites of sarcoma, and various times from diagnosis to ensure measures that are developed or existing validated measures reflect issues important to patients and will therefore be sensitive enough to detect change. The final recommendation is for clinicians and researchers to take a more standard approach in the administration of outcome measures and report this more thoroughly; the criteria described to assess quality in this review could act as a guide.

## Figures and Tables

**Table 1:** Quality assessment criteria.

Category	Description
C1. Sample	Are details provided about the total population who are eligible to take part during the study period in enough detail that a response rate can be calculated?
C2. Valid measure	Is the measure valid for the included population, i.e., has been validated for the age and there is a valid translation available if used outside of the language it was originally developed?
C3. Purpose	Is it clear what the PROM measures?
C4. Domains	If the PROM is known to have domain scores, have these been accurately reported?
C5. Scoring	Have details of how the total and/or domain score are interpreted?
C6. Administration	Are details of the administration of the PROM included; as a minimum this needs to state the mode (interview, postal, or online)?
C7. Missing data	Have statistical approaches for dealing with missing data been explicitly stated?
C8. Nonparticipants	Has a comparison been made between those who participated and those who refused?

**Table 2:** Overview of study aims and methodology.

First author	Year	Country of origin	Study aims	Study design	Setting	Time focus	Quality score
Sugarbaker et al. [94]	1982	USA	To compare assessment of QOL between AMP versus LSS	Interventional^1^	Single	During treatment	Q2/3^3^
Weddington et al. [102]	1985	USA	To determine if LSS had better psychological outcomes than AMP in extremity sarcoma	Observational	Single	Follow-up	Q1
Postma et al. [80]	1992	Netherlands	To compare QOL in lower limb BT for LSS versus AMP	Observational	Single	Follow-up	Q2
Rougraff et al. [86]	1994	USA	To compare long-term outcomes for survivors of OS between LSS, AMP, and disarticulation at the hip	Observational	Multicentre	Long-term survivor	Q3
Sammallahti et al. [88]	1995	Finland	To describe the defences AYA OS survivors use	Observational	Single	Follow-up	Q2
Christ et al. [36]	1996	USA	To explore patterns of adjustment of long-term survivors of lower limb BT	Observational	Single	Long-term survivor	Q2
Felder-Puig et al. [46]	1998	Austria	To evaluate psychosocial adjustment, assess age-appropriate achievements, and identify problems in AYA with BT	Observational	Single	Follow-up	Q2/3
Davis et al. [41]	1999	Canada	To compare levels of disability between patients treated with LSS versus AMP	Observational	Single	Follow-up	Q2/3
Hillmann et al. [56]	1999	Germany	To evaluate the effect of rotationplasty, AMP, and LSS on QOL	Observational	Single	Follow-up	Q2/3
Davis et al. [40]	2000	Canada	To identify predictors of functional outcomes after LSS for STS	Observational	Single	Follow-up	Q2
Veenstra et al. [101]	2000	Netherlands	To assess the medium and long-term effects on QOL after rotationplasty	Observational	Multicentre	Follow-up	Q2
Eiser et al. [44]	2001	UK	To compare QOL to population norms and the differences between AMP versus LSS	Observational	Single	Follow-up	Q2/3
Malo et al. [64]	2001	Canada	To understand the impact of successful LSS for BT on patients' function	Observational	Multicentre	Follow-up	Q2/3
Rodl et al. [85]	2002	Germany	To evaluate QOL in patients at least 10 years after rotationplasty	Observational	Single	Long-term survivor	Q3
Servaes et al. [90]	2003	Netherlands	To investigate the prevalence and predictors of fatigue in patients with BT and STS	Longitudinal	Single	Follow-up	Q1
Marchese et al. [65]	2004	USA	To conduct a pilot study to examine the relationship between physical function and QOL in AYA survivors of OS	Pilot study	Single	Follow-up	Q2
Nagarajan et al. [69]	2004	USA	To assess function and QOL in long-term childhood survivors of lower limb BT	Observational	Multicentre	Long-term survivor	Q2
Zahlten-Hinguranage et al. [107]	2004	Germany	To determine the predictors of whether QOL is high for patients with AMP or LSS	Observational^2^	Single	Follow-up	Q2
Koopman et al. [61]	2005	Netherlands	To investigate QOL and coping strategies in children at 3 and 8 years after the end of treatment	Longitudinal	Single	Follow-up	Q1
Tabone et al. [96]	2005	France	To assess the factors that impact on QOL in patients who had childhood BT	Observational	Multicentre	Follow-up	Q2
Gerber et al. [51]	2006	USA	To evaluate function and performance in adult survivors of child and adolescent sarcoma	Observational	Single	Follow-up	Q2
Hoffmann et al. [59]	2006	Germany	To determine the impact of surgery on QOL and function in long-term survivors after acetabulum resection	Observational	Single	Long-term survivor	Q2/3
Hopyan et al. [60]	2006	Australia	To determine whether children with AMP or rotationplasty were more physically active, functionally satisfied, and less psychosocial cost than those with LSS	Observational	Single	Long-term survivor	Q1
Marchese et al. [66]	2006	USA	Hypothesised that limited range of movement in children and adolescents who had LSS would have impaired functional mobility affecting QOL	Observational	Multicentre	Follow-up	Q3
Schreiber et al. [89]	2006	Canada	To evaluate how functional disability impacts on QOL of patients with extremity STS 1 year after surgery	Observational	Multicentre	Follow-up	Q2/3
Thijssens et al. [99]	2006	Netherlands	To investigate whether STS survivors had different QOL than a reference group and identify predictors of QOL and stress response	Observational	Single	Follow-up	Q2/3
Wiener et al. [104]	2006	USA	To determine the prevalence of psychological distress and posttraumatic stress symptoms in childhood sarcoma survivors	Observational	Single	Long-term survivor	Q1
Akahane et al. [28]	2007	Japan	To compare QOL for patients with OS around the knee between rotationplasty, LSS, and AMP	Observational	Multicentre	Follow-up	Q2/3
Aksnes et al. [29]	2007	Norway	To compare QOL, fatigue and mental distress in childhood survivors of BT to those with Hodgkin's disease, testicular cancer, and normative data	Observational	Multicentre	Long-term survivor	Q1
Ginsberg et al. [52]	2007	USA	To compare QOL and functional outcomes of AYA survivors of lower limb BT after AMP, LSS, and rotationplasty	Observational	Multicentre	Follow-up	Q2/3
Beck et al. [32]	2008	USA	To compare functional outcomes and QOL following internal or external hemipelvectomy	Observational	Single	Follow-up	Q2/3
Davidge et al. [38]	2009	Canada	To examine the impact of preoperative outcome expectations with postoperative function and QOL	Observational	Single	Follow-up	Q2/3
Hinds et al. [57]	2009	USA	To evaluate the ability of adolescents at the time of diagnosis to self-report their QOL	Observational	Single	Diagnosis	Q1
Hinds et al. [58]	2009	USA	To assess the effect of treatment on children and adolescents QOL at the time of diagnosis, during and after treatment, and assess for differences in sex and age	Longitudinal	Multicentre	From diagnosis to follow-up	Q1
Nagarajan et al. [70]	2009	USA	To describe global function in childhood BT survivors, evaluate variables that may predict global function, and explore associations with QOL	Observational	Multicentre	Long-term survivor	Q2
Yonemoto et al. [106]	2009	Japan	To describe psychosocial outcomes of long-term child and adolescent survivors of OS	Observational	Single	Long-term survivor	Q2
Barrera et al. [30]	2010	Canada	To examine the impact of surgery and gender on sexual function in AYA survivors of lower limb BT	Observational	Single	Long-term survivor	Q1
Bekkering et al. [34]	2010	Netherlands	To compare QOL in children and AYA following surgery for BT around the knee joint of the leg with healthy controls	Observational	Multicentre	Follow-up	Q2
Robert et al. [84]	2010	USA	To compare psychosocial and functional outcomes of LSS and AMP in OS survivors	Observational	Single	Follow-up	Q2
Granda-Cameron et al. [54]	2011	USA	To examine symptom distress and QOL in newly diagnosed patients with sarcoma	Observational	Single	Diagnosis	Q2
Nagarajan et al. [71]	2011	USA	To evaluate survival, medical, and psychosocial outcomes and health status of survivors of childhood OS	Observational	Multicentre	Long-term survivor	Q2/3
Paredes et al. [74]	2011	Portugal	To examine change in QOL through diagnosis to treatment, and analyse predictors of QOL	Longitudinal	Single	Diagnosis and during treatment	Q1
Paredes et al. [73]	2011	Portugal	To understand how patients adjust to a sarcoma diagnosis at difference phases of the disease experience	Observational	Multicentre	Diagnosis, treatment, and follow-up	Q3
Expósito Tirado et al. [45]	2011	Spain	To compare QOL and physical function in young people with LSS versus AMP	Observational	Single	Long-term survivor	Q1
Barrera et al. [31]	2012	Canada	To investigate QOL in AYA survivors of lower limb BT as function of type of surgery, age, and gender	Observational	Single	Long-term survivor	Q1
Bekkering et al. [33]	2012	Netherlands	To evaluate QOL, functional ability, and physical activity during the first 2-years following surgery	Longitudinal	Multicentre	During treatment and follow-up	Q2
Forni et al. [49]	2012	Italy	To gain more knowledge on the QOL and experience of patients treated by rotationplasty and identify factors related to disability	Observational	Single	Follow-up	Q2
Han et al. [55]	2012	China	To investigate the QOL of patients with BT after surgery	Longitudinal	Single	During treatment and follow-up	Q1
Paredes et al. [76]	2012	Portugal	To determine if greater perceived social support is related to lower anxiety and depressions and better QOL, and explore differences at different phases of disease	Observational	Multicentre	Diagnosis, treatment, and follow-up	Q1
Paredes et al. [75]	2012	Portugal	To assess the emotional adjustment to diagnosis and treatment, and identify demographic and clinical variables predictive of adjustment	Longitudinal	Multicentre	Diagnosis and during treatment	Q1
Reichardt et al. [82]	2012	Canada, USA, Germany, France, Italy, Netherlands, Spain, UK, Sweden	To describe utility weights in metastatic sarcoma and explore QOL according to predefined health states	Observational	Multicentre	Metastatic disease	Q3
Smorti [92]	2012	Italy	To assess adolescents' expectations of the future after bone cancer treatment and to investigate the relationship between expectations of the future, resilience and coping strategies	Observational	Single	Follow-up	Q1
Sun et al. [95]	2012	China	To assess QOL after surgical treatment for BT and assess risk factors for improving physical and mental QOL	Longitudinal	Single	During treatment and follow-up	Q2/3
Teall et al. [98]	2012	Canada	To examine perceived social support and benefit finding with respect to surgical intervention, gender, and age; to compare these to normative values; and to examine the relationship between social and psychological outcomes and sexual functioning	Observational	Multicentre	Long-term survivor	Q1
Marina et al. [67]	2013	USA	To compare health status and participation restriction outcomes longitudinally for extremity sarcoma survivors to determine whether the trajectory over time varies as a function of tumour location	Longitudinal	Multicentre	Follow-up	Q3
Mason et al. [68]	2013	USA	To determine if there is a difference in QOL related to AMP or LSS	Observational	Single	Follow-up	Q2
Liu et al. [63]	2014	China	To explore the correlation between functional status and QOL in patients with lower limb BT	Observational	Multicentre	Follow-up	Q2/3
Ostacoli et al. [72]	2014	Italy	To compare QOL and anxiety and depression in the early stages of treatment compared to those with common types of cancer	Observational	Multicentre	During treatment	Q2/3
van Riel et al. [100]	2014	Netherlands	To assess self-perception and QOL of adolescents during or up to 3 months after adjuvant treatment for BT	Observational	Single	During treatment and follow-up	Q2
Chan et al. [35]	2015	Singapore	To describe QOL, symptom burden, and medication use in adult sarcoma patients	Observational	Single	During treatment	Q1
Custers et al. [37]	2015	Netherlands	To assess QOL, distress, and fear of cancer recurrence or progression in patients with GIST	Observational	Single	During treatment	Q1
Furtado et al. [50]	2015	UK	To describe physical function, QOL, and pain after AMP	Observational	Multicentre	Follow-up	Q2
Gradl et al. [53]	2015	Germany	To assess long-term QOL, functional performance, and psychosocial aspects after rotationplasty	Observational	Single	Long-term survivor	Q2
Rivard et al. [83]	2015	Canada	To document functional outcome and QOL in relation to wound complication rates	Observational	Single	During treatment and follow-up	Q2
Shchelkova and Usmanova [91]	2015	Russia	To investigate QOL and the relation to disease in patients with malignant BT	Observational	Single	ns	Q3
Stish et al. [93]	2015	USA	To assess patient-reported functional and QOL outcomes in survivors of ES	Observational	Single	Long-term survivor	Q1
Tang et al. [97]	2015	Australia	To identify the prevalence, trajectory, and determinants of distress and characterise sources of stress in patients with extremity sarcoma	Longitudinal	Single	Diagnosis and during treatment	Q2
Fidler et al. [48]	2015	UK	To investigate the long-term risks of adverse outcomes in 5-year survivors of childhood bone sarcoma	Observational	National	Long-term survivor	Q2/3
Davidson et al. [39]	2016	Canada	To estimate the change in QOL between diagnosis and 1-year after surgery	Longitudinal	Single	Diagnosis and during treatment	Q1
Dressler et al. [42]	2016	USA	To analyse of long-term QOL outcomes for patients with GIST	Observational	Single	Follow-up	Q1
Edelmann et al. [43]	2016	USA	To examine neurocognitive, neurobehavioural, emotional, and QOL outcomes in long-term survivors of childhood OS	Observational	Single	Long-term survivor	Q2
Leiser et al. [62]	2016	Switzerland, Germany	To evaluate clinical outcomes for children with RMS treated with pencil beam scanning, assess QOL, and identify prognostic factors for tumour control	Longitudinal	Multicentre	During treatment and follow-up	Q2
Phukan et al. [77]	2016	USA	To report QOL and functional outcomes after sacrectomy for malignant BT	Observational	Single	Follow-up	Q1
Poort et al. [79]	2016	Netherlands	To determine the prevalence of severe fatigue in patients with GIST, the impact on QOL, psychosocial and physical function, and the association with tyrosine kinase inhibitor use	Observational	Single	Follow-up	Q2
Weiner et al. [103]	2016	UK	To explore the extent of which child, adolescents, and their family engaged with psychological screening and whether they report concerns during the follow-up appointments	Feasibility	Single	ns	Q2/3
Bekkering et al. [108]	2017	Netherlands	To assess the course of QOL over time between 2 and 5 years or more after surgery	Longitudinal	Multicentre	Long-term survivor	Q2
Fernandez-Pineda et al. [47]	2017	USA	To compare QOL and social role attainment between extremity sarcoma and healthy control	Observational	Single	Long-term survivor	Q2
Podleska et al. [78]	2017	Germany	To gain insight into patients' QOL after isolated limb perfusion and long-term survival	Observational	Single	Follow-up	Q2/3
Ranft et al. [81]	2017	Germany, Netherlands, Austria	To gather information on long-term outcome of ES, and look for prognostic factors for these outcomes	Observational	Multicentre	Follow-up	Q1
Saebye et al. [87]	2017	Denmark	To identify tumour- and patient-related factors associated with QOL after LSS for STS	Observational	Multicentre	Follow-up	Q2
Wong et al. [105]	2017	Canada	To examine how treatment-related toxicities affect QOL of patients with retroperitoneal sarcoma	Observational	Single	Follow-up	Q3

AMP: amputation; AYA: adolescents and young adults; BT: bone tumour; ES: Ewing sarcoma; GIST: gastrointestinal stromal tumour; LSS: limb-sparing surgery; ns: not stated; OS: osteosarcoma; QOL: quality of life; RMS: rhabdomyosarcoma; STS: soft tissue sarcoma. ^1^Patient reported outcome measured as part of a clinical trial but reported independent to the trial results as it was an observational study. ^2^Described by the authors as a “qualitative study.” ^3^Quality rating includes 50% in both Q2 and Q3, so these were classified as both and rated as borderline poor.

**Table 3:** Participant characteristics.

First author	Participants (response %)^1^	Type of sarcoma	Site	Age at study (years)	Gender male (%)
Sugarbaker et al. [94]	21 (91)	STS	Extremity	ns	ns
Weddington et al. [102]	33 (67)	BT, STS	Extremity	Range 15–71	45
Postma et al. [80]	33 (92)	BT	LL	Range 13–56	55
Rougraff et al. [86]	29 (13)	OS	LL	ns	66
Sammallahti et al. [88]	16 (100)	OS	All	Range 21–31	50
Christ et al. [36]	45 (69)	BT	LL	Range 17–34	58
Felder-Puig et al. [46]	60 (55)	BT	Extremity	M 23.5 (sd 4.3)	57
Davis et al. [41]	12 (92)	BT, STS	LL	M 34.4 (sd 11.6)	67
Hillmann et al. [56]	65 (97)	BT	LL	Range 11–24^3^	62
Davis et al. [40]	172 (76)	STS	LL	M 51 (sd 15.2)	51
Veenstra et al. [101]	33 (97)	BT	LL	Range 16–50	55
Eiser et al. [44]	37 (93)	BT	LL	Range 12–47	57
Malo et al. [64]	53 (95)	BT	LL	M 36.7 (sd 18.3)	53
Rodl et al. [85]	22^2^	BT	LL	Range 18–49	ns
Servaes et al. [90]	170 (75)	BT, STS	ns	Range 18–65	53
Marchese et al. [65]	18 (64)	OS	LL	Range 10–27	44
Nagarajan et al. [69]	528 (84)	BT	LL/pelvis	M 34.8 (sd 19.5)	49
Zahlten-Hinguranage et al. [107]	124 (66)	BT	LL	Range 14–76	63
Koopman et al. [61]	18 (90)	BT	Extremity	Range 12–23	72
Tabone et al. [96]	37^2^	BT	All	Range 10–18	68
Gerber et al. [51]	32 (40)	BT	ns	M 35.4 (sd 10.6)	53
Hoffmann et al. [59]	45 (71)	BT	Pelvis	Range 16.1–83.2	64
Hopyan et al. [60]	45 (83)	BT	LL	Range 10–39	49
Marchese et al. [66]	68^2^	BT	LL	Range 10–26	56
Schreiber et al. [89]	100 (90)	STS	Extremity	Range 18–86	56
Thijssens et al. [99]	39 (95)	STS	Extremity	Range 15–78	41
Wiener et al. [104]	34 (41)	BT, STS	ns	M 17 (sd 5)	53
Akahane et al. [28]	21 (72)	OS	LL	Range 8–69	81
Aksnes et al. [29]	57 (76)	BT	Extremity	Male M 34 (sd 9.4), female M 27 (sd 4.8)	54
Ginsberg et al. [52]	91^2^	BT	LL	M 20.1 (sd 5.7)	53
Beck et al. [32]	97 (94)	BT	Pelvis/femur	IQR 33.3–66.5^3^	68
Davidge et al. [38]	157 (100)	STS	Extremity	Range 16.1–87	62
Hinds et al. [57]	39 (93)	OS	All	Range 13–23	54
Hinds et al. [58]	66 (93)	OS	All	Range 5–23.5	55
Nagarajan et al. [70]	528 (84)	BT	LL	M 34.8 (sd 5.8)	49
Yonemoto et al. [106]	30 (55)	OS	All	Range 7–17^3^	37
Barrera et al. [30]	28 (39)	BT	LL	M 25.1 (sd 4.5)	50
Bekkering et al. [34]	81 (92)	BT	Knee	M 16.9 (sd 4.2)	49
Robert et al. [84]	57 (57)	OS	Extremity	Range 16.1–52	35
Granda-Cameron et al. [54]	11 (65)	BT, STS	ns	M 44.5 (sd 13.7)	36
Nagarajan et al. [71]	733 (68)	OS	All	Range 13–51	52
Paredes et al. [74]	36 (88)	BT, STS	All	Range 18–72	53
Paredes et al. [73]	142^2^	BT, STS	All	M 48.3 (sd 16.4)^4^, M 48.1 (sd 17.7), M 48.3 (sd 18.5)	56
Expósito Tirado et al. [45]	17 (44)	OS, ES	Extremity	Range 20–25	41
Barrera et al. [31]	28 (40)	BT	LL	M 25.1 (sd 4.5)	50
Bekkering et al. [33]	44 (90)	BT	Knee	M 14.9 (sd 4.8)^3^	61
Forni et al. [49]	20 (67)	BT	Femur	Range 17–38	60
Han et al. [55]	120 (100)	BT	LL	M 14.1 (sd 4.6)^3^	66
Paredes et al. [76]	151^2^	BT, STS	All	M 47.5 (sd 17)^4^, M 44.9 (sd 16.9), M 46.9 (sd 18.1)	56
Paredes et al. [75]	36 (88)	BT, STS	All	M 40.5 (sd 16)	53
Reichardt et al. [82]	116^2^	BT, STS	All	Range 18.5–83.4	41
Smorti [92]	32 (80)	BT	ns	Range 11–20	56
Sun et al. [95]	344 (97)	BT	LL	M 18.7 (sd 4.9)	57
Teall et al. [98]	28 (40)	BT	LL	Range 18–32	50
Marina et al. [67]	1094^2^	BT, STS	Extremity	Range 10–53	Unclear
Mason et al. [68]	82 (82)	BT	LL	14–19.9	52
Liu et al. [63]	94 (88)	BT	LL	M 22.8 (sd 9.7)	45
Ostacoli et al. [72]	56^2^	STS	All	M 53.5 (sd 14.1)	50
van Riel et al. [100]	10^2^	BT	All	Range 12–17	60
Chan et al. [35]	79 (98)	BT, STS, GIST	ns	M 57.3 (sd 15.2)	58
Custers et al. [37]	54 (63)	GIST	GI	Range 21–84	54
Furtado et al. [50]	100 (40)	BT, STS	LL	Range 9–91	60
Gradl et al. [53]	12 (86)	BT	LL	M 33 (sd 11)	58
Rivard et al. [83]	45 (87)	STS	All	Range 24–83	78
Shchelkova and Usmanova [91]	82^2^	BT	ns	Range 18–67	57
Stish et al. [93]	74 (56)	ES	All	Range 12.2–83.8	62
Tang et al. [97]	76 (75)	BT, STS	Extremity	Range 16–86	59
Fidler et al. [48]	411 (81)	BT	All	Range 7.5–76.8	84
Davidson et al. [39]	220 (38)	STS	Extremity	M 54.4 (sd 16.6)	59
Dressler et al. [42]	36 (52)	GIST	GI	Range 42–89	56
Edelmann et al. [43]	80 (67)	OS	ns	M 38.9 (sd 7.1)	58
Leiser et al. [62]	83 (91)	Rhabdomyosarcoma	ns	Range 0.8–15.5^3^	55
Phukan et al. [77]	33 (73)	BT	Sacrum	Range 23–77^5^	58
Poort et al. [79]	89 (75)	GIST	GI	Range 21–86	58
Weiner et al. [103]	21 (91)	BT	ns	Range 9–18	52
Bekkering et al. [108]	20 (45)	BT	Knee	M 22.3 (sd 4.0)	50
Fernandez-Pineda et al. [47]	206 (63)	BT, STS	Extremity	Range 19.4–65.1	52
Podleska et al. [78]	26 (96)	STS	LL	Range 12–73	54
Ranft et al. [81]	614 (47)	ES	All	ns	56
Saebye et al. [87]	128 (67)	STS	LL	IQR 47–70	45
Wong et al. [105]	48^2^	STS	Retroperitoneal	Range 38–82^3^	54

BT: bone tumour; ES: Ewing sarcoma; GIST: gastrointestinal stromal tumour; IQR: interquartile range; LL: lower limb; M: mean; ns: not stated; OS: osteosarcoma; sd: standard deviation; STS: soft tissue sarcoma. ^1^Calculated from interpreting the information reported in the paper not necessarily what the authors report. ^2^Not enough detail reported to be able to calculate a response rate. ^3^Age at diagnosis; age at study not reported. ^4^Age reported for each group: diagnosis, treatment, and follow-up. ^5^The age reported in the text is different to the age reported in the table (range 33–77). ^6^Reported for the whole cohort (*n* = 664) not just the 411 respondents of the patient-reported outcome.

**Table 4:** Longitudinal outcomes^1^.

First author	Comparator^2^	Quality of life^3^	Domains^3^	Mental health	Others
Davidge et al. [38]	Time: before surgery vs. after surgery	+			+ adjustment to normal life
Hinds et al. [58]	Time: diagnosis to end of treatment	+	PF, EF		
Granda-Cameron et al. [54]	Time: cycles 1–8 of chemotherapy	ns			
Paredes et al. [74]	Time: diagnosis to treatment	+ −	GH PF		
Paredes et al. [73]	Time: diagnosis to follow-up			=	
Bekkering et al. [33]	Time: 3 to 12 months after surgery^4^ 12 to 24 months after surgery^4^	+ +	BT specific, PCS PCS		
Han et al. [55]	Time: before surgery to 6 months after surgery 6 to 12 months after surgery	+ =			
Paredes et al. [75]	Time: diagnosis to treatment			=	
Sun et al. [95]	Time: treatment to 1 year after treatment	ns			
Rivard et al. [83]	Time: before surgery vs. 12 months after surgery	+			
Leiser et al. [62]	Time: treatment to 2 years after surgery	+			
Bekkering et al. [108]	Time: 3 to >60 months after surgery	+	PCS		
Wong et al. [105]	Time: before treatment to 5 years after treatment	ns			

BT: bone tumour; EF: emotional function; GH: global health; ns: significance not specified; OS: osteosarcoma; PCS: physical component score; PF: physical function. ^1^Minus (−): poorer in comparison; plus (+): better; equals (=): no difference. ^2^Direction of significance, i.e., better or worse, based on the first comparator, or the last time point if a longitudinal comparison. ^3^Result based on overall or summary scores; if these were not provided, result at domain score level was provided (QLQ-C30 functional scale only). ^4^Based on SF-36 and bone tumour-specific measure results reflecting the comparison across the whole sample.

**Table 5:** Comparison between different types of surgery^1^.

First author	Comparator^2^	Quality of life^3^	Domains^3^	Mental health	Self-worth	Others
Sugarbaker et al. [94]	AMP vs. LSS			=		
Weddington et al. [102]	AMP vs. LSS			=		
Postma et al. [80]	AMP vs. LSS	=		=	=	
Rougraff et al. [86]	AMP vs. LSS vs. hip disarticulation			=		
Christ et al. [36]	AMP vs. LSS			−	=	
Davis et al. [41]	AMP vs. LSS	−^4^	PF			
Hillmann et al. [56]	Rotationplasty vs. LSS	+	Role			
Eiser et al. [44]	AMP vs. LSS	=^4^				=body image
Nagarajan et al. [69]	AMP vs. LSS	=				
Zahlten-Hinguranage et al. [107]	AMP vs. LSS	=				=life satisfaction
Hoffmann et al. [59]	Hip disarticulation vs. AMP vs. LSS	^5^				
Hopyan et al. [60]	Rotationplasty, AMP vs. LSS	=				
Akahane et al. [28]	Rotationplasty, AMP vs. LSS	=				
Ginsberg et al. [52]	Rotationplasty, AMP vs. LSS	=				
Beck et al. [32]	Internal vs. external hemipelvectomy	=				
Barrera et al. [30]	AMP vs. LSS			−	+	=sexual function
Robert et al. [84]	AMP vs. LSS	=			=	=body image =social support
Expósito Tirado et al. [45]	AMP vs. LSS	=				
Barrera et al. [31]	AMP vs. LSS	=				
Teall et al. [98]	AMP vs. LSS					=social support and benefit finding
Mason et al. [68]	AMP vs. LSS	−				
Bekkering et al. [108]	AMP vs. LSS	−	PCS			

AMP: amputation; LSS: limb-sparing surgery; PCS: physical component score; PF: physical function. ^1^Minus (−): poorer in comparison; plus (+): better; equals (=): no difference. ^2^Direction of significance, i.e., better or worse, based on the first comparator, or the last time point if a longitudinal comparison. ^3^Result based on overall or summary scores; if these were not provided, result at domain score level was provided (QLQ-C30 functional scale only). ^4^Total and/or summary scores can be calculated with the measure used, but this was not reported. ^5^Text is unclear, and data presented in an appendix are no longer available.

**Table 6:** Comparison to a reference value^1,2^.

First author	Quality of life^3^	Domains^3^
Veenstra et al. [101]	−^4^	PF, RP
Eiser et al. [44]	−^4^	PF, RP, SF, vitality, pain, GH
Malo et al. [64]	−^4^	PF, RP
Rodl et al. [85]	=	
Koopman et al. [61]	−(1997) +(2002)	MF, autonomy Cognition, SF, NE
Gerber et al. [51]	=	
Hoffmann et al. [59]	=	
Thijssens et al. [99]	−^3^	PF, RP
Aksnes et al. [29]	−	
Bekkering et al. [34]	− +	PCS MCS
Paredes et al. [74]	^4^-	PF, RP, GH, SF
Barrera et al. [31]	^5^	
Forni et al. [49]	−^4^ +	PF MH
Reichardt et al. [82]	ns	
Sun et al. [95]	ns	
Liu et al. [63]	−	
van Riel et al. [100]	−	PWB, autonomy, SE, SS
Gradl et al. [53]	+^4^	RS, MH, vitality
Fidler et al. [48]	−	
Edelmann et al. [43]	−^4^	PF, GH
Leiser et al. [62]	=	
Weiner et al. [103]	=	
Fernandez-Pineda et al. [47]	−	PCS
Podleska et al. [78]	=	
Ranft et al. [81]	−	PCS

GH: global health; MCS: mental component score; MF: motor function; MH: mental health; NE: negative emotion; ns: significance not specified; PCS: physical component score; PF: physical function; PWB: physical wellbeing; RP: role-physical; RS: role-social; SE: school environment; SF: social function; SS: social support/peers. ^1^Minus (−): poorer in comparison; plus (+): better; equals (=): no difference. ^2^Reference values either supplied with the measure or collected from noncancer controls as part of the study. ^3^Result based on overall or summary scores; if these were not provided, result at domain score level was provided (QLQ-C30 functional scales only). ^4^Total and/or summary scores can be calculated with the measure used, but this was not reported. ^5^Three quality of life measures used, all giving different results.

**Table 7:** Comparison to other cancer types^1^.

First author	Comparator^2^	Quality of life^3^	Domains^3^	Mental health	Others
Aksnes et al. [29]	Hodgkin's disease Testicular cancer	− −	PCS PCS	= =	=fatigue =fatigue
Hinds et al. [57]	Acute myeloid leukaemia	−			
Nagarajan et al. [71]	Survivors of other cancers			-	
Ostacoli et al. [72]	Common cancers	=		-	
Podleska et al. [78]	Other cancer patients	+			

PCS: physical component score. ^1^Minus (−): poorer in comparison; plus (+): better; equals (=): no difference. ^2^Direction of significance, i.e., better or worse, based on the first comparator, or the last time point if a longitudinal comparison. ^3^Result based on overall or summary scores; if these were not provided, result at domain score level was provided (QLQ-C30 functional scale only).

**Table 8:** QOL measured by the SF-36^1^.

First author	Comparator^2^	Quality of life^3^	Domains^3^
Davis et al. [41]	AMP vs. LSS	−^5^	PF
Veenstra et al. [101]	Reference values^4^	−^5^	PF, RP
Eiser et al. [44]	AMP vs. LSS Reference values	=^5^ −^5^	PF, RP, SF, vitality, pain, GH
Malo et al. [64]	Reference values	−^5^	PF, RP
Gerber et al. [51]	Reference values	=	
Hopyan et al. [60]	Rotationplasty, AMP vs. LSS	=	
Thijssens et al. [99]	Reference values	−^5^	PF, RP
Akahane et al. [28]	Rotationplasty, AMP vs. LSS	=	
Aksnes et al. [29]	Reference values Hodgkin's disease Testicular cancer	− − −	PCS PCS
Ginsberg et al. [52]	Rotationplasty, AMP vs. LSS	=	
Bekkering et al. [34]	Reference values^7^	− +	PCS MCS
Expósito Tirado et al. [45]	AMP vs. LSS	=	
Barrera et al. [31]	AMP vs. LSS Reference values	=^6^	
Bekkering et al. [33]	Time: 3 to 12 months after surgery^7^ 12 to 24 months after surgery^7^	+ +	BT specific, PCS PCS
Forni et al. [49]	Reference values	−^5^ +	PF MH
Han et al. [55]	Time: before surgery to 6 months after surgery 6 to 12 months after surgery	+ =	
Sun et al. [95]	Time: treatment to 1 year after treatment Reference values	ns ns	
Liu et al. [63]	Reference values	−	
Gradl et al. [53]	Reference values	^+5^	RS, MH, vitality
Rivard et al. [83]	Time: before surgery vs. 12 months after surgery	+	
Shchelkova and Usmanova [91]	GCT vs. OS GCT vs. CS	−^5^ −^5^	PF, MH, GH, SF PF, SF
Fidler et al. [48]	Reference values	−	
Edelmann et al. [43]	Reference values	−^5^	PF, GH
Poort et al. [79]	Severe fatigue vs. none	−	
Bekkering et al. [108]	AMP vs. LSS Time: 3 to >60 months after surgery	− +	PCS PCS
Fernandez-Pineda et al. [47]	Reference values	−	PCS
Ranft et al. [81]	Reference values	−	PCS

AMP: amputation; BT: bone tumour; GCT: giant cell tumour; GH: global health; LSS: limb-sparing surgery; MH: mental health; ns: significance not specified; OS: osteosarcoma; PCS: physical component score; PF: physical function; RP: role-physical; RS: role-social; SF: social function. ^1^Minus (−): poorer in comparison; plus (+): better; equals (=): no difference. ^2^Direction of significance, i.e., better or worse, based on the first comparator, or the last time point if a longitudinal comparison. ^3^Result based on overall or summary scores; if these were not provided, result at domain score level was provided (QLQ-C30 functional scale only). ^4^Reference values either supplied with the measure or collected from noncancer controls as part of the study. ^5^Total and/or summary scores can be calculated with the measure used, but this was not reported. ^6^Three quality of life measures used, all giving different results. ^7^Based on SF-36 and bone tumour-specific measure results reflecting the comparison across the whole sample.

**Table 9:** QOL measured by the QLQ-C30^1^.

First author	Comparator^2^	Quality of life^3^	Domains^3^
Hillmann et al. [56]	Rotationplasty vs. LSS	+	Role
Veenstra et al. [101]	Reference values^4^	−^5^	PF, RP
Rodl et al. [85]	Reference values	=	
Zahlten-Hinguranage et al. [107]	AMP vs. LSS	=	
Hoffmann et al. [59]	Hip disarticulation vs. AMP vs. LSS Reference values	=^6^	
Paredes et al. [74]	Time: diagnosis to treatment Reference value	+^5^ − −	GH PF PF, RP, GH, SF
Barrera et al. [31]	AMP vs. LSS Reference values	= ^7^	
Reichardt et al. [82]	Metastatic STS vs. metastatic BT Reference values	ns ns	
Custers et al. [37]	High vs. low fear of recurrence	−	
Shchelkova and Usmanova [91]	GCT vs. OS GCT vs. CS	−^5^ −^5^	PF, MH, GH, SF PF, SF
Tang et al. [97]	Distress vs. no distress	−	
Poort et al. [79]	Severe fatigue vs. none	−	
Podleska et al. [78]	Other cancer patients Reference values	+ =	
Wong et al. [105]	Time: before treatment to 5 years after treatment	ns	

AMP: amputation; BT: bone tumour; GCT: giant cell tumour; GH: global health; LSS: limb-sparing surgery; MH: mental health; ns: significance not specified; OS: osteosarcoma; PF: physical function; RP: role-physical; SF: social function; STS: soft tissue sarcoma. ^1^Minus (−): poorer in comparison; plus (+): better; equals (=): no difference. ^2^Direction of significance, i.e., better or worse, based on the first comparator, or the last time point if a longitudinal comparison. ^3^Result based on overall or summary scores; if these were not provided, result at domain score level was provided (QLQ-C30 functional scale only). ^4^Reference values either supplied with the measure or collected from noncancer controls as part of the study. ^5^Total and/or summary scores can be calculated with the measure used, but this was not reported. ^6^Text is unclear, and data presented in an appendix are no longer available. ^7^Three quality of life measures used, all giving different results.

## Supplementary Materials

Supplementary Materials The supplemental files contain detailed tables of information referred to in the text of the manuscript.
