# Supplementary material for: A Critical Review of the Impact of Sarcoma on Psychosocial Wellbeing
Source: Sarcoma. 2019 Feb 17;2019:9730867. doi: 10.1155/2019/9730867 (PMC6397984; doi:10.1155/2019/9730867)
Supplement: Supplementary Materials — The supplemental files contain detailed tables of information referred to in the text of the manuscript. [file 9730867.f1.docx]

APPENDIX

Table A1: Search terms

|  | Search terms | | |
| --- | --- | --- | --- |
| Population | Sarcoma  Bone tumour  Gastrointestinal stromal tumour (GIST)   - Ewing sarcoma - Osteosarcoma - Chondrosarcoma - Rhabdomyosarcoma | | |
| Outcome | **Quality of life**  Satisfaction  Happiness  Health status  Well-being  Lived experience | **Psychological**  Emotional distress  Cognitive functioning  Depression  Anxiety  Adaptation  Resilience  Coping  Behaviour  Adjustment  Emotions | **Social**  Sexual function  Sexuality  Employment  Education  Social support  Friends  Marriage  Relationships  Finances |

Table A2: Quality assessment

| **First author** | **C1** | **C2** | **C3** | **C4** | **C5** | **C6** | **C7** | **C8** | **Total^1^ (%)** | **Quality score^2^** |
| --- | --- | --- | --- | --- | --- | --- | --- | --- | --- | --- |
| Sugarbaker et al. [94] | Yes | Yes | Yes | Yes | No | No | No | No | 50 | Q2/3 |
| Weddington et al. [102] | Yes | Yes | Yes | Yes | Yes | Yes | No | Yes | 88 | Q1 |
| Postma et al. [80] | Yes | Yes | Yes | Yes | Yes | No | No | No | 63 | Q2 |
| Rougraff et al. [86] | Yes | Yes | Yes | No | No | No | No | No | 38 | Q3 |
| Sammallahti et al. [88] | Yes | Yes | Yes | Yes | No | Yes | No | No | 63 | Q2 |
| Christ et al. [36] | Yes | Yes | Yes | Yes | No | Yes | No | No | 63 | Q2 |
| Felder-Puig et al. [46] | Yes | Yes | Yes | Yes | No | No | No | No | 50 | Q2/3 |
| Davis et al. [41] | Yes | Yes | Yes | No | Yes | No | No | No | 50 | Q2/3 |
| Hillman et al. [56] | No | Yes | Yes | Yes | Yes | No | No | No | 50 | Q2/3 |
| Davis et al. [40] | Yes | Yes | Yes | Yes | Yes | No | No | No | 63 | Q2 |
| Veenstra et al. [101] | Yes | No | Yes | Yes | Yes | Yes | No | No | 63 | Q2 |
| Eiser et al. [44] | Yes | No | Yes | Yes | Yes | No | No | No | 50 | Q2/3 |
| Malo et al. [64] | Yes | Yes | Yes | Yes | No | No | No | No | 50 | Q2/3 |
| Rodl et al. [85] | No | Yes | Yes | No | No | No | No | No | 25 | Q3 |
| Servaes et al. [90] | Yes | Yes | Yes | Yes | Yes | Yes | No | No | 75 | Q1 |
| Marchese et al. [65] | Yes | No | Yes | Yes | No | Yes | No | Yes | 63 | Q2 |
| Nagarajan et al. [69] | Yes | Yes | Yes | Yes | No | No | No | Yes | 63 | Q2 |
| Zahlten-Hinguranage et al. [107] | Yes | No | Yes | Yes | Yes | Yes | No | No | 63 | Q2 |
| Koopman et al. [61] | Yes | Yes | Yes | Yes | Yes | Yes | No | No | 75 | Q1 |
| Tabone et al. [96] | No | Yes | Yes | Yes | Yes | Yes | No | No | 63 | Q2 |
| Gerber et al. [51] | Yes | Yes | Yes | No | No | Yes | No | Yes | 63 | Q2 |
| Hoffman et al. [59] | Yes | No | Yes | Yes | Yes | No | No | No | 50 | Q2/3 |
| Hopyan et al. [60] | Yes | Yes | Yes | Yes | No | Yes | No | Yes | 75 | Q1 |
| Marchese et al. [66] | No | No | Yes | Yes | No | Yes | No | No | 38 | Q3 |
| Schreiber et al. [89] | Yes | No | Yes | Yes | Yes | Yes | No | No | 50 | Q2/3 |
| Thijssens et al. [99] | No | No | Yes | Yes | Yes | Yes | No | No | 50 | Q2/3 |
| Wiener et al. [104] | Yes | Yes | Yes | Yes | Yes | Yes | No | Yes | 88 | Q1 |
| Akahane et al. [28] | Yes | No | Yes | Yes | Yes | No | No | No | 50 | Q2/3 |

| Table A2 *cont.* |  |  |  |  |  |  |  |  |  |  |
| --- | --- | --- | --- | --- | --- | --- | --- | --- | --- | --- |
| **First author** | **C1** | **C2** | **C3** | **C4** | **C5** | **C6** | **C7** | **C8** | **Total^1^ (%)** | **Quality score^2^** |
| Aksnes et al. [29] | Yes | Yes | Yes | Yes | Yes | Yes | No | Yes | 88 | Q1 |
| Ginsberg et al. [52] | No | Yes | Yes | Yes | No | Yes | No | No | 50 | Q2/3 |
| Beck et al. [32] | Yes | Yes | Yes | Yes | No | No | No | No | 50 | Q2/3 |
| Davidge et al. [38] | Yes | No | Yes | Yes | Yes | No | No | No | 50 | Q2/3 |
| Hinds et al. [57] | Yes | Yes | Yes | Yes | Yes | Yes | Yes | No | 88 | Q1 |
| Hinds et al. [58] | Yes | Yes | Yes | Yes | Yes | Yes | Yes | No | 88 | Q1 |
| Nagarajan et al. [70] | Yes | Yes | Yes | Yes | No | No | No | Yes | 63 | Q2 |
| Yonemoto et al. [106] | Yes | Yes | Yes | No | No | Yes | No | Yes | 63 | Q2 |
| Barrera et al. [30] | Yes | Yes | Yes | Yes | Yes | No | No | Yes | 75 | Q1 |
| Bekkering et al. [34] | Yes | No | Yes | Yes | Yes | Yes | No | No | 63 | Q2 |
| Robert et al. [84] | Yes | Yes | Yes | Yes | No | Yes | No | No | 63 | Q2 |
| Granda-Cameron et al. [54] | Yes | Yes | Yes | Yes | Yes | Yes | No | No | 75 | Q2 |
| Nagarajan et al. [71] | Yes | Yes | Yes | No | No | No | No | Yes | 50 | Q2/3 |
| Paredes et al. [74] | Yes | Yes | Yes | Yes | Yes | Yes | Yes | No | 88 | Q1 |
| Paredes et al. [73] | No | No | Yes | Yes | Yes | No | No | No | 38 | Q3 |
| Expósito Tirado et al. [45] | Yes | Yes | Yes | Yes | Yes | Yes | No | No | 75 | Q1 |
| Barrera et al. [31] | Yes | Yes | Yes | Yes | Yes | Yes | No | Yes | 88 | Q1 |
| Bekkering et al. [33] | Yes | Yes | Yes | Yes | Yes | No | No | No | 63 | Q2 |
| Forni et al. [49] | Yes | No | Yes | Yes | Yes | Yes | No | No | 63 | Q2 |
| Han et al. [55] | Yes | Yes | Yes | Yes | Yes | Yes | No | No | 75 | Q1 |
| Paredes et al. [76] | No | Yes | Yes | Yes | Yes | Yes | Yes | No | 75 | Q1 |
| Paredes et al. [75] | Yes | Yes | Yes | Yes | Yes | Yes | Yes | No | 88 | Q1 |
| Reichardt et al. [82] | No | Yes | Yes | Yes | No | No | No | No | 38 | Q3 |
| Smorti [92] | Yes | Yes | Yes | Yes | Yes | Yes | No | Yes | 88 | Q1 |
| Sun et al. [95] | Yes | No | Yes | Yes | No | Yes | No | No | 50 | Q2/3 |
| Teall et al. [98] | Yes | Yes | Yes | Yes | Yes | Yes | No | Yes | 88 | Q1 |
| Marina et al. [67] | No | No | Yes | No | No | No | No | No | 13 | Q3 |
| Mason et al. [68] | Yes | Yes | Yes | Yes | No | Yes | No | No | 63 | Q2 |
| Liu et al. [63] | Yes | No | Yes | No | Yes | Yes | No | No | 50 | Q2/3 |
| Ostacoli et al. [72] | No | Yes | Yes | Yes | Yes | No | No | No | 50 | Q2/3 |
| van Riel et al. [100] | No | Yes | Yes | Yes | Yes | Yes | No | No | 63 | Q2 |

| Table A2 *cont.* |  |  |  |  |  |  |  |  |  |  |
| --- | --- | --- | --- | --- | --- | --- | --- | --- | --- | --- |
| **First author** | **C1** | **C2** | **C3** | **C4** | **C5** | **C6** | **C7** | **C8** | **Total^1^ (%)** | **Quality score^2^** |
| Chan et al. [35] | Yes | Yes | Yes | Yes | Yes | Yes | No | No | 75 | Q1 |
| Custers et al. [37] | Yes | Yes | Yes | Yes | Yes | No | Yes | Yes | 88 | Q1 |
| Furtado et al. [50] | Yes | No | Yes | Yes | Yes | Yes | No | No | 63 | Q2 |
| Gradl et al. [53] | Yes | Yes | Yes | Yes | Yes | No | No | No | 63 | Q2 |
| Rivard et al. [83] | Yes | Yes | Yes | Yes | Yes | No | No | No | 63 | Q2 |
| Shchelkova and Usmanova [91] | No | Yes | Yes | Yes | No | No | No | No | 38 | Q3 |
| Stish et al. [93] | Yes | Yes | Yes | No | Yes | Yes | No | Yes | 75 | Q1 |
| Tang et al. [97] | Yes | No | Yes | Yes | Yes | Yes | No | No | 63 | Q2 |
| Fidler et al. [48] | Yes | No | Yes | Yes | No | Yes | No | No | 50 | Q2/3 |
| Davidson et al. [39] | Yes | Yes | Yes | Yes | Yes | No | No | Yes | 75 | Q1 |
| Dressler et al. [42] | Yes | Yes | Yes | Yes | No | Yes | No | Yes | 75 | Q1 |
| Edelmann et al. [43] | Yes | Yes | Yes | No | No | Yes | No | Yes | 63 | Q2 |
| Leiser et al. [62] | Yes | Yes | Yes | Yes | Yes | No | No | No | 63 | Q2 |
| Phukan et al. [77] | Yes | Yes | Yes | Yes | Yes | No | Yes | No | 75 | Q1 |
| Poort et al. [79] | Yes | Yes | Yes | No | Yes | No | No | Yes | 63 | Q2 |
| Weiner et al. [103] | Yes | Yes | Yes | No | No | Yes | No | No | 50 | Q2/3 |
| Bekkering et al. [108] | Yes | Yes | Yes | Yes | Yes | No | No | No | 63 | Q2 |
| Fernandez-Pineda et al. [47] | Yes | Yes | Yes | Yes | Yes | No | No | No | 63 | Q2 |
| Podleska et al. [78] | Yes | No | Yes | Yes | Yes | No | No | No | 50 | Q2/3 |
| Ranft et al. [81] | Yes | Yes | Yes | Yes | No | Yes | No | Yes | 75 | Q1 |
| Saebye et al. [87] | Yes | Yes | Yes | No | Yes | No | No | Yes | 63 | Q2 |
| Wong et al. [105] | No | Yes | Yes | No | Yes | No | No | No | 38 | Q3 |

^1^Percentage of the number of the specified criteria that were reported as specified (Table 1)

^2^Q1: >75% of the criteria; Q2: 50-74%; Q3: <50%

C1 – Sample: Are details provided about the total population who are eligible to take part during the study period in enough detail that a response rate can be calculated?

C2 - Valid measure: Is the measure valid for the included population, i.e. has been validated for the age and there is a valid translation available if used outside of the language it was originally developed?

C3 – Purpose: Is it clear what the PROM measures?

C4 – Domains: If the PROM is known to have domain scores, have these been accurately reported?

C5 – Scoring: Have details of how the total and/or domain score are interpreted?

C6 – Administration: Are details of the administration of the PROM included; as a minimum this needs to state the mode (interview, postal, online)?

C7 - Missing data: Have statistical approaches for dealing with missing data explicitly stated?

C8 - Non-participants: Has a comparison been made between those who participated and those who refused?

Table A3: Psycho-social domains and outcome measures used^1^

| **First author** | **Psychosocial domain(s)** | **Outcome measure(s)** |
| --- | --- | --- |
| Sugarbaker et al. [94] | Mental wellbeing | Psychosocial Adjustment to Illness Scale (PAIS) |
| Weddington et al. [102] | Mental wellbeing | Hopkins Symptom Check List (HSCL)  Beck Depression Inventory (BDI)  Profile of Mood States scale (POMS)  Global Adjustment to Illness scale (GAIS)  Schedule for Affective Disorders and Schizophrenia – Lifetime version (SADS-L) |
| Postma et al. [80] | QOL  Mental wellbeing  Self-worth | Visual Analogue Scale (VAS)  HSCL  Rosenberg Self-esteem scale (RSES) |
| Rougraff et al. [86] | Mental wellbeing | Brief Symptom Inventory (BSI)  PAIS |
| Sammallahti et al. [88] | Mental wellbeing | Symptom Check List (SCL-90)  Defense Style Questionnaire (DSQ) |
| Christ et al. [36] | Mental wellbeing  Self-worth | BSI  Centre for Epidemiological Study Depression Scale (CES-D)  RSES |
| Felder-Puig et al. [46] | Mental wellbeing  Self-worth  Satisfaction with life | Subjective Well-Being (3 domains)  State-Trait Anxiety Inventory (1 domain)  Frankfurt Self-Concept Scales (10 domains)  Questionnaire on Life Goals and Satisfaction (3 items) |
| Davis et al. [41] | QOL  Adjustment to normal life | Short Form-36 (SF-36)  Reintegration to Normal Living scale (RNL) |
| Hillman et al. [56] | QOL | EORTC Quality of Life Questionnaire Core module (QLQ-C30) |
| Davis et al. [40] | QOL | SF-36 |
| Veenstra et al. [101] | QOL  Social support  Body image | QLQ-C30  SF-36  Social support List-Interactions  Social Support List-Discrepancies  EORTC Breast Cancer Module (QLQ-BR23; 1 question only) |
| Eiser et al. [44] | QOL  Body image | SF-36  Body Image Instrument |
| Malo et al. [64] | QOL | SF-36 |
| Rodl et al. [85] | QOL  Satisfaction with life | QLQ-C30  Life Satisfaction Questionnaire (FLZ) |
| Servaes et al. [90] | QOL  Mental wellbeing  Fatigue  Optimism | QLQ-C30  SCL-90  Cancer Acceptance Scale (CAS)  Life Orientation Test (LOT); |
| Marchese et al. [65] | QOL | SF-36 |
| Nagarajan et al. [69] | QOL | Quality of Life for Cancer Survivors (QOL-CS) |
| Zahlten-Hinguranage et al. [107] | QOL  Satisfaction with life | QLQ-C30  FLZ |

| Table A3 *cont.* |  |  |
| --- | --- | --- |
| **First author** | **Psychosocial domain(s)** | **Outcome measure(s)** |
| Koopman et al. [61] | QOL  Coping | TNO-AZL-Child Quality of Life (TACQOL)  TNO-AZL Adult Quality of Life (TAAQOL)  Utrecht coping list adolescents questionnaire (UCLA) |
| Tabone et al. [96] | QOL | Children Health Questionnaire (CHQ) |
| Gerber et al. [51] | QOL  Social wellbeing | SF-36  Leisure Satisfaction Measure (LSM)  Goldberg Scale of Vocational Development (GSVD)  Occupational Performance History Interview |
| Hoffman et al. [59] | QOL | QLQ-C30 |
| Hopyan et al. [60] | QOL | SF-36 |
| Marchese et al. [66] | QOL | SF-36 |
| Schreiber et al. [89] | QOL  Adjustment to normal life | European Quality of Life Questionnaire (EQ5D)-VAS  RNLI |
| Thijssens et al. [99] | QOL  Mental wellbeing | RAND-36 (identical to the SF-36 but scored differently)  Impact of Event Scale (IES) |
| Wiener et al. [104] | Mental wellbeing | BSI  Structured clinical interview for DSM-IV (SCID)  IES |
| Akahane et al. [28] | QOL | SF-36 |
| Aksnes et al. [29] | QOL  Mental wellbeing  Fatigue | SF-36  Hospital Anxiety and Depression Scale (HADS)  Fatigue Questionnaire |
| Ginsberg et al. [52] | QOL | SF-36 |
| Beck et al. [32] | QOL | Linear Analog Self-Assessment (LASA) |
| Davidge et al. [38] | QOL | EQ5D-VAS |
| Hinds et al. [57] | QOL | Pediatric Quality of Life Inventory (PedsQL) |
| Hinds et al. [58] | QOL | PedsQL  PedsQL Cancer Module |
| Nagarajan et al. [70] | QOL  Adjustment to normal life | QOL-CS  RNLI |
| Yonemoto et al. [106] | Mental wellbeing  Social support  Family function | IES  Posttraumatic Growth Inventory (PTGI)  Social Support Questionnaire (SSQ)  Adaptability, Partnership, Growth, Affection, and Resolve (APGAR) |
| Barrera et al. [30] | Mental wellbeing  Self-worth  Sexual function | CES-D  Adult Self Perception Profile (ASPP)  Brief Sexual Function Questionnaire for Men (BSFQ-M)  Brief Index of Sexual Functioning for Women (BISF-W) |
| Bekkering et al. [34] | QOL | TACQOL  TAAQOL  SF-36 |

| Table A3 *cont.* |  |  |
| --- | --- | --- |
| **First author** | **Psychosocial domain(s)** | **Outcome measure(s)** |
| Robert et al. [84] | QOL  Body image  Self-worth  Social support | Quality of Life – Cancer Specific Scale (QOL-CSS)  Amputee Body Image Scale (ABIS)  Index of Self-Esteem  Sarason's Social Support Questionnaire |
| Granda-Cameron et al. [54] | QOL | Functional Assessment of Cancer Therapy – General (FACT-G) |
| Nagarajan et al. [71] | Mental wellbeing | BSI |
| Paredes et al. [74] | QOL | QLQ-C30 |
| Paredes et al. [73] | Mental wellbeing | HADS |
| Expósito Tirado et al. [45] | QOL | SF-36 |
| Barrera et al. [31] | QOL | QLQ-C30  SF-36  Health Utilities Index (HUI2, HUI3) |
| Bekkering et al. [33] | QOL | TACQOL  TAAQOL  SF-36  Bt-DUX (bone tumour-specific measure of QOL) |
| Forni et al. [49] | QOL | SF-36 |
| Han et al. [55] | QOL | SF-36 |
| Paredes et al. [76] | QOL  Mental wellbeing  Social support | World Health Organisation Quality of Life Assessment (WHOQOL-Bref)  HADS  Medical Outcome Study Social Support Survey (MOS-SSS) |
| Paredes et al. [75] | Mental wellbeing  Coping | HADS  Brief Cope |
| Reichardt et al. [82] | QOL | QLQ-C30  EQ5D-VAS |
| Smorti [92] | Resilience  Coping  Expectations of the future | Ego-Resiliency Scale  Coping Strategies Indicator  Expectations for Future Scale |
| Sun et al. [95] | QOL | SF-36 |
| Teall et al. [98] | Mental wellbeing  Benefit finding  Social support  Self-worth  Sexual functioning | CES-D  Benefit Finding Scale (BFS)  MOS-SSS  ASPP  BSFQ-M  BISF-W |
| Marina et al. [67] | Mental wellbeing | BSI |
| Mason et al. [68] | QOL  Mental wellbeing | Quality of Life Questionnaire (QLQ)  Minnesota Multiphasic Personality Inventory (MMPI) |
| Liu et al. [63] | QOL  Adjustment to normal life | SF-36  RNLI |
| Ostacoli et al. [72] | QOL  Mental wellbeing | FACT-G  HADS |

| Table A3 *cont.* |  |  |
| --- | --- | --- |
| **First author** | **Psychosocial domain(s)** | **Outcome measure(s)** |
| van Riel et al. [100] | QOL  Self-worth | KIDSCREEN-52  Self-Perception Profile for Adolescents (SPPA) |
| Chan et al. [35] | QOL  Mental wellbeing | FACT-G  Rotterdam Symptom Checklist  Beck Anxiety Inventory |
| Custers et al. [37] | QOL  Mental wellbeing  Fear of recurrence | QLQ-C30  HADS  IES  Cancer Worry Scale  Fear of Cancer Recurrence Inventory |
| Furtado et al. [50] | QOL | QOL-CS |
| Gradl et al. [53] | QOL | SF-36 |
| Rivard et al. [83] | QOL | SF-36 |
| Shchelkova and Usmanova [91] | QOL | SF-36  QLQ-C30  BM22 (EORTC bone metastases module) |
| Stish et al. [93] | QOL | PedsQL |
| Tang et al. [97] | QOL  Mental wellbeing | QLQ C-30  Depression Anxiety and Stress Scale 21 (DASS21)  Shame and Stigma Scale (SSS) |
| Fidler et al. [48] | QOL | SF-36 |
| Davidson et al. [39] | QOL | EQ5D |
| Dressler et al. [42] | QOL | Gastrointestinal Quality of Life Index (GIQLI) |
| Edelmann et al. [43] | QOL  Mental wellbeing | SF-36  BSI |
| Leiser et al. [62] | QOL | PedsQL |
| Phukan et al. [77] | QOL | Patient Reported Outcome Measurement Information System (PROMIS) measures |
| Poort et al. [79] | QOL  Mental wellbeing  Self-efficacy  Fatigue | QLQ-C30  SF-36  HADS  Self-Efficacy Scale  Fatigue Catastrophizing Scale |
| Weiner et al. [103] | QOL  Mental wellbeing | EQ5D  Strengths and Difficulties Questionnaire  Short Mood and Feelings Questionnaire  Distress Thermometer |
| Bekkering et al. [108] | QOL | TACQOL  TAAQOL  SF36  Bt-DUX |
| Fernandez-Pineda et al. [47] | QOL | SF-36 |
| Podleska et al. [78] | QOL | QLQ-C30 |
| Ranft et al. [81] | QOL  Mental wellbeing  Self-worth | SF-36  BSI  RSES |
| Saebye et al. [87] | QOL | QLQ-C30 |
| Wong et al. [105] | QOL | QLQ-C30 |

QOL: quality of life

^1^ Studies may measure other aspects of wellbeing, i.e. physical function but only psychosocial aspects are reported in this review

Table A4: Summary of results^1^

| **First author** | **Comparator^2^** | **Quality of life^3^** | **Domains^3^** | **Mental**  **Health** | **Self-worth** | **Other** |
| --- | --- | --- | --- | --- | --- | --- |
| Sugarbaker et al. [94] | AMP vs. LSS |  |  | = |  |  |
| Weddington et al. [102] | AMP vs. LSS |  |  | = |  |  |
| Postma et al. [80] | AMP vs. LSS | = |  | = | = |  |
| Rougraff et al. [86] | AMP vs. LSS vs. hip disarticulation |  |  | = |  |  |
| Sammallahti et al. [88] | Reference values^4^ |  |  | = |  |  |
| Christ et al. [36] | AMP vs. LSS  Reference values |  |  | -  = | = |  |
| Felder-Puig et al. [46] | None |  |  |  |  |  |
| Davis et al. [41] | AMP vs. LSS | ^5^ - | PF |  |  |  |
| Hillman et al. [56] | Rotationplasty vs. LSS | + | Role |  |  |  |
| Davis et al. [40] | None |  |  |  |  |  |
| Veenstra et al. [101] | Reference values | ^5^ - | PF, RP |  |  | = social support |
| Eiser et al. [44] | AMP vs. LSS  Reference values | ^5^ =  ^5^ - | PF,RP, SF, vitality, pain, GH |  |  | = body image |
| Malo et al. [64] | Reference values | ^5^ - | PF, RP |  |  |  |
| Rodl et al. [85] | Reference values | = |  |  |  | = life satisfaction |
| Servaes et al. [90] | None |  |  |  |  |  |
| Marchese et al. [65] | None |  |  |  |  |  |
| Nagarajan et al. [69] | AMP vs. LSS | = |  |  |  |  |
| Zahlten-Hinguranage et al. [107] | AMP vs. LSS | = |  |  |  | = life satisfaction |
| Koopman et al. [61] | Reference values (1997)  Reference values (2002) | -  + | MF, autonomy  Cognition, SF, NE |  |  |  |
| Tabone et al. [96] | None |  |  |  |  |  |
| Gerber et al. [51] | Reference values | = |  |  |  | + leisure satisfaction |
| Hoffman et al. [59] | Hip disarticulation vs. AMP vs. LSS  Reference values | ^6^  = |  |  |  |  |
| Hopyan et al. [60] | Rotationplasty, AMP vs. LSS | = |  |  |  |  |
| Marchese et al. [66] | None |  |  |  |  |  |
| Schreiber et al. [89] | None |  |  |  |  |  |

| Table A4 *cont.* |  |  |  |  |  |  |
| --- | --- | --- | --- | --- | --- | --- |
| **First author** | **Comparator^2^** | **Quality of life^3^** | **Domains^3^** | **Mental**  **Health** | **Self-worth** | **Other** |
| Thijssens et al. [99] | Reference values | ^5^ - | PF, RP |  |  |  |
| Wiener et al. [104] | Reference values |  |  | - |  |  |
| Akahane et al. [28] | Rotationplasty, AMP vs. LSS | = |  |  |  |  |
| Aksnes et al. [29] | Reference values  Hodgkins disease  Testicular cancer | -  -  - | PCS  PCS | +  =  = |  | - fatigue  = fatigue  = fatigue |
| Ginsberg et al. [52] | Rotationplasty, AMP vs. LSS | = |  |  |  |  |
| Beck et al. [32] | Internal vs. external hemipelvectomy | = |  |  |  |  |
| Davidge et al. [38] | Time: pre vs. post-surgery | + |  |  |  | + adjustment to normal life |
| Hinds et al. [57] | Acute myeloid leukaemia | - |  |  |  |  |
| Hinds et al. [58] | Time: diagnosis to end of treatment | + | PF, EF |  |  |  |
| Nagarajan et al. [70] | None |  |  |  |  |  |
| Yonemoto et al. [106] | None |  |  |  |  |  |
| Barrera et al. [30] | Male vs. female  AMP vs. LSS |  |  | =  - | =  + | + sexual function  = sexual function |
| Bekkering et al. [34] | Reference values^7^ | -  + | PCS  MCS |  |  |  |
| Robert et al. [84] | AMP vs. LSS | = |  |  | = | = body image  = social support |
| Granda-Cameron et al. [54] | Time: cycles 1-8 of chemotherapy | ns |  |  |  |  |
| Nagarajan et al. [71] | Reference values  Survivors of other cancers |  |  | -  - |  |  |
| Paredes et al. [74] | Time: diagnosis to treatment  Reference value | ^5^ +  -  - | GH  PF  PF, RP, GH, SF |  |  |  |
| Paredes et al. [73] | Time: diagnosis to follow up |  |  | = |  |  |
| Expósito Tirado et al. [45] | AMP vs. LSS | = |  |  |  |  |
| Barrera et al. [31] | AMP vs. LSS  Reference values | =  ^8^ |  |  |  |  |

| Table A4 *cont.* |  |  |  |  |  |  |
| --- | --- | --- | --- | --- | --- | --- |
| **First author** | **Comparator^2^** | **Quality of life^3^** | **Domains^3^** | **Mental**  **Health** | **Self-worth** | **Other** |
| Bekkering et al. [33] | Time: 3 to 12 months post-surgery^7^  12 to 24 months post-surgery^7^ | +  + | BT specific, PCS  PCS |  |  |  |
| Forni et al. [49] | Reference values | ^5^ –  + | PF  MH |  |  |  |
| Han et al. [55] | Time: Pre to 6 months post-surgery  6 to 12 months post-surgery | +  = |  |  |  |  |
| Paredes et al. [76] | None |  |  |  |  |  |
| Paredes et al. [75] | Time: diagnosis to treatment |  |  | = |  |  |
| Reichardt et al. [82] | Metastatic STS vs. metastatic BT  Reference values | ns  ns |  |  |  |  |
| Smorti [92] | Reference values |  |  |  |  | + expectations for the future  - resilience  = coping |
| Sun et al. [95] | Time: treatment to 1 year after treatment  Reference values | ns  ns |  |  |  |  |
| Teall et al. [98] | AMP vs. LSS  Reference values |  |  | - | = | = social support and benefit finding (both comparators) |
| Marina et al. [67] | None |  |  |  |  |  |
| Mason et al. [68] | AMP vs. LSS | - |  |  |  |  |
| Liu et al. [63] | Reference values | - |  |  |  |  |
| Ostacoli et al. [72] | Common cancers | = |  | - |  |  |
| van Riel et al. [100] | Reference values | - | PWB, autonomy, SE, SS |  | = |  |
| Chan et al. [35] | GIST vs. non-GIST | + |  | + |  |  |
| Custers et al. [37] | High vs. low fear of recurrence | - |  | - |  |  |
| Furtado et al. [50] | Level of AMP | = |  |  |  |  |
| Gradl et al. [53] | Reference values | ^5^ + | RS, MH, vitality |  |  |  |
| Rivard et al. [83] | Time: pre vs. 12 months post-surgery | + |  |  |  |  |

| Table A4 *cont.* |  |  |  |  |  |  |
| --- | --- | --- | --- | --- | --- | --- |
| **First author** | **Comparator^2^** | **Quality of life^3^** | **Domains^3^** | **Mental**  **Health** | **Self-worth** | **Other** |
| Shchelkova and Usmanova [91] | GCT vs. OS  GCT vs. CS | ^5^ -  ^5^ - | PF, MH, GH, SF  PF, SF |  |  |  |
| Stish et al. [93] | None |  |  |  |  |  |
| Tang et al. [97] | Distress vs. no distress | - |  | - |  | - shame and stigma |
| Fidler et al. [48] | Reference values | - |  |  |  |  |
| Davidson et al. [39] | None |  |  |  |  |  |
| Dressler et al. [42] | None |  |  |  |  |  |
| Edelmann et al. [43] | Reference values | ^5^ - | PF, GH |  |  |  |
| Leiser et al. [62] | Time: treatment to 2 years post  Reference values (at 2 years post) | +  = |  |  |  |  |
| Phukan et al. [77] | Total vs. partial sacrectomy | - | GP | = |  |  |
| Poort et al. [79] | Severe fatigue vs. none | - |  | - |  | - self efficacy |
| Weiner et al. [103] | Reference values | = |  | = |  |  |
| Bekkering et al. [108] | AMP vs. LSS  Time: 3 to >60 months after surgery | -  + | PCS  PCS |  |  |  |
| Fernandez-Pineda et al. [47] | Reference values | - | PCS |  |  |  |
| Podleska et al. [78] | Other cancer patients  Reference values | +  = |  |  |  |  |
| Ranft et al. [81] | Reference values | - | PCS | - | - |  |
| Saebye et al. [87] | None |  |  |  |  |  |
| Wong et al. [105] | Time: pre-treatment to 5 years post | ns |  |  |  |  |

AMP: amputation; BT: bone tumour; CS: chondrosarcoma; EF: emotional function; GCT: giant cell tumour; GH: global health; GP: global item-physical; LSS: limb sparing surgery; MCS: mental component score; MF: motor function; MH: mental health; NE: negative emotion; ns: significance not specified; OS: osteosarcoma; PCS: physical component score; PF: physical function; RP: role-physical; PWB: physical wellbeing; RS: role-social; SE: school environment; SF: social function; SS: social support/peers; STS: soft tissue sarcoma

^1^ minus (-): poorer in comparison; plus (+): better; equals (=): no difference

^2^ Direction of significance, i.e. better or worse, based on the first comparator, or the last time point if a longitudinal comparison

^3^ Result based on overall or summary scores; if these were not provided, result at domain score level was provided (QLQ-C30 functional scale only)

^4^ Reference values either supplied with the measure or collected from non-cancer controls as part of the study

^5^ Total and/or summary scores can be calculated with the measure used but this was not reported

^6^ Text is unclear and data presented in an appendix is no longer available

^7^ Based on SF36 and bone tumour specific measure results reflecting the comparison across the whole sample

^8^Three quality of life measures used, all giving different results
